# Development of Eye Position Dependency of Slow Phase Velocity during Caloric Stimulation

**DOI:** 10.1371/journal.pone.0051409

**Published:** 2012-12-12

**Authors:** Christopher J. Bockisch, Elham Khojasteh, Dominik Straumann, Stefan C. A. Hegemann

**Affiliations:** 1 Department of Otorhinolaryngology, Head & Neck Surgery, University Hospital Zürich, Zürich, Switzerland; 2 Department of Neurology, University Hospital Zürich, Zürich, Switzerland; 3 Department of Ophthalmology, University Hospital Zürich, Zürich, Switzerland; 4 Zürich Centre for Integrative Human Physiology (ZIHP), University of Zürich, Zürich, Switzerland; McMaster University, Canada

## Abstract

The nystagmus in patients with vestibular disorders often has an eye position dependency, called Alexander’s law, where the slow phase velocity is higher with gaze in the fast phase direction compared with gaze in the slow phase direction. Alexander’s law has been hypothesized to arise either due to adaptive changes in the velocity-to-position neural integrator, or as a consequence of processing of the vestibular-ocular reflex. We tested whether Alexander’s law arises only as a consequence of non-physiologic vestibular stimulation. We measured the time course of the development of Alexander’s law in healthy humans with nystagmus caused by three types of caloric vestibular stimulation: cold (unilateral inhibition), warm (unilateral excitation), and simultaneous bilateral bithermal (one side cold, the other warm) stimulation, mimicking the normal push-pull pattern of vestibular stimulation. Alexander’s law, measured as a negative slope of the velocity versus position curve, was observed in all conditions. A reversed pattern of eye position dependency (positive slope) was found <10% of the time. The slope often changed with nystagmus velocity (cross-correlation of nystagmus speed and slope was significant in 50% of cases), and the average lag of the slope with the speed was not significantly different from zero. Our results do not support the hypothesis that Alexander’s law can only be observed with non-physiologic vestibular stimulation. Further, the rapid development of Alexander’s law, while possible for an adaptive mechanism, is nonetheless quite fast compared to most other ocular motor adaptations. These results suggest that Alexander’s law may not be a consequence of a true adaptive mechanism.

## Introduction

Alexander’s law describes how the slow phase velocity of nystagmus varies with eye position [1], where the slow phase velocity is faster when looking in the direction of the fast phase of nystagmus than in the slow phase direction. This behaviour can be observed in most patients with an acute unilateral vestibular deficit.

Robinson et al [2] and Hess [3] proposed that changes in the velocity-to-position neural integrator are responsible for Alexander’s law. The integration of the ocular motor velocity command into a position command is necessary to counter centripetal elastic forces of the eye plant [4], [5]. If neural integration is diminished, the fixation command is insufficient to keep a normal eye from drifting towards a central position, producing gaze-evoked nystagmus whose velocity increases with eccentricity. If gaze-evoked nystagmus is combined with the vestibular nystagmus, drift velocity in one gaze direction is reduced, but is increased in the opposite direction.

Robinson et al. (1984) investigated the time course of the development of the eye position dependency in three subjects during caloric induced nystagmus and found that it first occurred 20–46 s after the onset of nystagmus [2]. With natural vestibular stimulation (real movements of the head in space on a turntable), the eye position dependency was small, and did not evolve over time, which led to the proposition that Alexander’s law is an adaptive response to un-natural vestibular stimulation. By ‘unnatural’, Robinson et al meant that a change in vestibular input from one side is not accompanied by the opposite change from the other side for 25 seconds. We will use the term non-physiologic to describe such stimulation patterns. If Alexander’s law is produced by changes in the neural integrator, it could be considered an adaptive response, since eye velocity will be reduced for some eye positions, thus aiding vision.

During normal yaw head turns, one horizontal semicircular canal is stimulated while the other is inhibited. During non-physiologic unilateral caloric stimulation, on the other hand, only one canal changes its tonic activity depending on the stimulus (increase with warm and decrease with cold stimulation). This unusual pattern of stimulation might be detected and lead to Alexander’s law.

In contrast, Doslak et al [6], [7] proposed that the rotational vestibular ocular reflex (rVOR) command has a gaze position component which leads to Alexander’s law. While this model can provide an account for Alexander’s law, it also predicts such effects for the normal VOR, which are not seen for head impulses [8], or 0.5 hz frequency head rotations [2].

We tested Robinson’s hypothesis that only non-physiologic velocity commands evoke Alexander’s law. We assumed that simultaneous irrigation at 44°C on one side (warm, excitatory) and 30°C (cold, inhibitory) on the contralateral side would produce a stimulation pattern similar to that produced by head rotations. If the intervestibular mismatch of non-physiologic stimulation would cause Alexander’s law, then we would expect that it does not develop during simultaneous bilateral bithermal stimulation or would at least be weaker compared to unilateral caloric stimulation. Contrary to this expectation, we found that Alexander’s law developed similarly in all conditions.

## Materials and Methods

### Subjects and equipment

Eleven subjects (8 male, 3 female) with no reported history of vestibular or oculomotor disorders participated, and each gave prior written consent after the experimental procedure was explained. The study was approved by the Ethics Committee of the Canton of Zurich, Switzerland, and was in accordance with the principles of the 1964 Declaration of Helsinki.

Subjects lay supine with the torso and head tilted up 30° to position the horizontal canals approximately vertical, which produces the strongest nystagmus. A neck cushion was used to reduce head motion. A 1.6 m×0.9 m screen was suspended from the ceiling, 1 meter from the subject and oriented perpendicular to the gaze line when subjects looked straight ahead. A mirror-galvanometer and laser under computer control projected a red target spot (∼0.25° diameter) onto the screen to control gaze direction.

Horizontal and vertical positions of the right eye were recorded at 220 Hz with head mounted video cameras (EyeSeeCam, Munich). The center of the pupil was determined by ellipse fits to thresholded images of each eye. A custom-made calibration of eye position was made by having subjects fixate targets at ±40°, ±30°, ±20°, ±10°, and 0° horizontally, and ±10° vertically. In practice, eye positions beyond ±30° could not be reliably measured and were excluded.

Warm (44°C), cold (30°C), and simultaneous bilateral bithermal caloric irrigation was performed at ∼230 ml/minute with ATMOS Variotherm plus systems. Caloric stimulation creates a temperature gradient along the lateral canals, introducing convection currents in the endolymph and movement of the cupula [9]. Cold stimulation produces utriculofugal flow of the endolymph, simulating a head rotation away from the irrigated ear and depressing activity in the vestibular nerve; warm stimulation has the opposite effect. Simultaneous bilateral bithermal stimulation (one side cold, the other warm; hereafter just called ‘bithermal’) thus approximates a normal horizontal head rotation. The altered temperature of caloric stimulation may also directly stimulate the vestibular nerve [10], [11].

### Procedure

Warm, cold, and bithermal stimulations were usually conducted on the same day in each subject. Each trial had 4 parts, with caloric stimulation in parts 3 and 4:

1. eye tracker calibration (35 sec)
2. baseline gaze holding (30 seconds)
3. Caloric stimulation (3 minutes):
4. Decline of nystagmus.

During baseline (part 2) and the first 2 minutes of stimulation, subjects were instructed to look in darkness at a pulsed target that moved every 4 seconds from 20° right to 20° left. The laser was pulsed (20 msec on, 2 sec off) so that we could direct the patient’s gaze direction without suppressing nystagmus. Two minutes after stimulation began, the targets ±20°, ±10°, and 0°, were presented in a pseudorandom order, in order to collect data at more fixation positions to allow for higher order fits through the velocity versus position curve. Prior to the first caloric stimulation trial, a 1 minute control trial with 5 target positions and no caloric stimulation was completed.

Caloric stimulation lasted 3 minutes, but we continued measurements as eye velocity declined until no nystagmus was noticeable or the subject became uncomfortable. Short breaks, of around 5 minute’s duration, were taken between recordings.

The stimulation order was constrained so that the direction of nystagmus changed for each trial. This required that the bithermal stimulation be either the first or last trial, and the unilateral stimulations be in the same ear. The first trial was warm for 3 subjects, cold for 4 subjects, and bithermal for 4 subjects. The unilateral stimulation was in the left ear for 5 subjects, and in the right ear for 6.

### Analysis

All analyses were performed with MATLAB^©^ (MathWorks Inc, Natick, MA, USA). Only horizontal eye movements were analyzed. We use the right-hand rule sign convention: looking left is positive and looking right is negative. To compare the data from different stimulation conditions, we converted the data to appear as if the subjects had right-sided excitation (warm right side, or cold left side), so slow phase eye movements were to the left.

Saccades were identified and removed with an interactive computer program that automatically detected saccades when velocity exceeded a threshold above the median eye velocity calculated over a 1 second window. With a window size this large there are more data points associated with slow phase movements than saccades, so the median is an estimate of the current slow-phase velocity, The threshold was typically set 20–30°/s from the median, depending upon the noise level. To ensure that saccadic components were not included, the initial 2 (∼10 msec) and final 5 samples (∼23 msec) were removed from each slow phase. The automatically-marked saccades could be manually adjusted and blink artifacts removed.

Nystagmus slow phases shorter than 50 msec were discarded, and slow phases longer than 100 msec were split into 2 or more parts of at least 50 msec. (This was done to ensure, for unbiased statistical analysis, that roughly the same number of data points occurred in each gaze direction.) For each of these slow phases we calculated the median position and velocity.

General estimates of the change of nystagmus velocity with eye position were made by linear regression of velocity versus position,
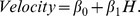

*β_0_* is the intercept, or velocity at gaze straight ahead, and *β_1_* is the slope parameter that describes how velocity changes which horizontal position, *H*.

We created ‘sliding’ fits, by fitting lines to 30 seconds of data, and then advancing the time period every 5 seconds. These fits gave us the most accurate estimates of the time of peak nystagmus velocity and slope. To investigate the correlation of the nystagmus velocity and slope, we made fits to blocks of data 16 seconds in duration (4 changes of flashing target position). We then cross-correlated the slopes and intercepts for each subject for the period when the nystagmus velocity was above 10% of the maximum velocity.

In patients suffering from spontaneous nystagmus due to an acute vestibular tone asymmetry, velocity varies with position in a non-linear fashion [12], [13], so we also wished to test if this was the case with nystagmus induced by calorics. After the first 2 minutes of stimulation, the flashing laser target alternated between 5 positions: ±20°, ±10°, and straight ahead, which allowed us to describe the change in velocity with horizontal eye position in more detail. We did this by fitting second order equations via linear regression to the horizontal velocity versus position data to the data collected 2–3 minutes after the start of stimulation,
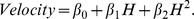



We occasionally recorded data long enough to observe a reversal of nystagmus, presumably an adaptation to the persistent caloric stimulation. We analyzed the 30 seconds of data around the peak reversal nystagmus for changes in velocity with eye position with linear fits.

To test for differences between warm, cold, and bithermal stimulations, we performed one-way repeated-measures analysis of variance (ANOVA) with SPSS (version 19), and if a significant effect was found, we then performed multiple comparison tests with Sidak’s correction. Correlations were performed with Spearmann’s rank correlation.

## Results

We first measured gaze holding prior to caloric stimulation by having subjects look to a flashing target that moved between positions of ±20°, ±10°, and straight ahead for 1 minute, and we fit second order equations to characterize the nystagmus velocity at straight ahead gaze (intercept), the first order change of the velocity with position (slope) and the 2^nd^ order change of velocity with position. The average best fit parameters were −0.18°/s (standard deviation = 0.43), −0.005 1/s (0.019), and −0.00004 1/°s (0.0003), none of which were significantly different from zero (p-values for difference from zero t-tests for the intercept, slope, and 2^nd^ order term were 0.18, 0.34, and 0.64, respectively). However, individual subjects could exhibit significant drift: 8/11 showed significant drift at gaze straight ahead (with a maximum of 0.8°/s), 6/11 had significant slope parameters, and 3/11 had significant quadratic components. The small amount of gaze evoked nystagmus is typical for healthy people [2], [14].

### Development of Alexander’s Law

**Figure 1:**
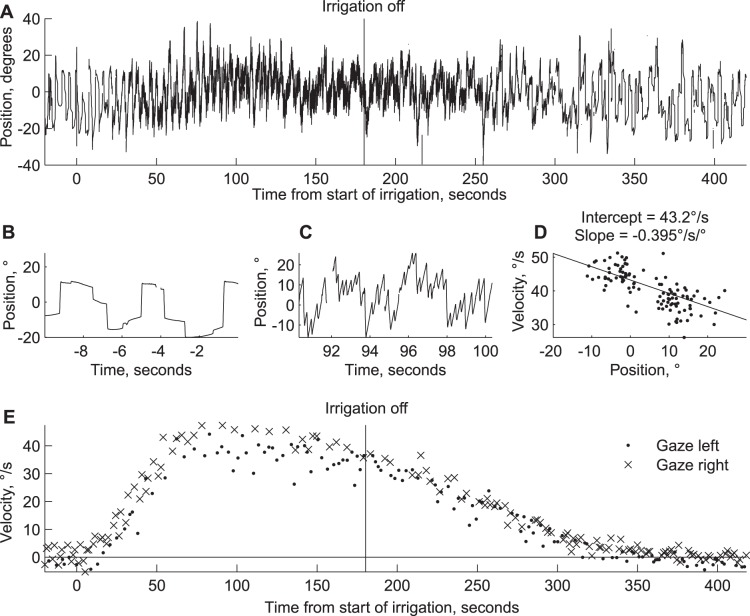
Example data. A. Horizontal eye position is shown for an experiment with bilateral, bithermal caloric stimulation. The subject looked into the direction of a flashing laser spot that first shifted between 20° left and right positions (time <120 s), and then the target shifted to targets at ±20°, ±10°, and 0° for the remainder of the experiment. B. Position traces before stimulation began, so little nystagmus is observed. C. Position traces from near the time of maximum velocity. D. Velocity is plotted versus position for 30 seconds of data selected at the time of maximum nystagmus. Each point is an individual slow phase. The best fit line and fitted parameters are also shown. E. The velocity of individual slow phases is shown, with different symbols used when subjects were looking left and right of straight ahead. For clarity, only 1 in 10 slow phases are shown.

**Figure 2:**
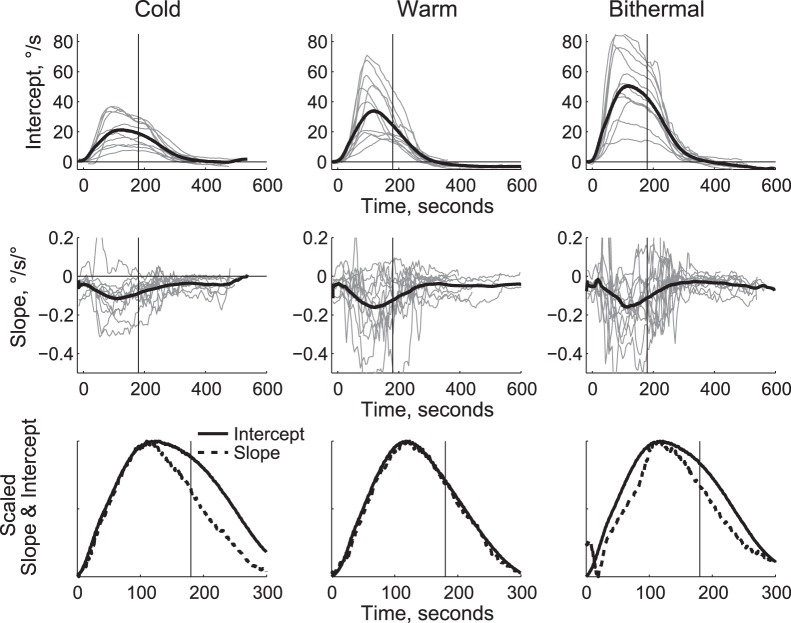
‘Sliding’ fits to all data. We made linear fits to the velocity versus position data, using 30 seconds of data, and shifting the center time every 5 seconds. The fitted intercepts (top row) and slopes (middle row) for velocity versus position are shown for each stimulation condition. The thin grey lines are individual subject data, and the thick black line is the mean. Vertical lines mark the time when caloric irrigation was stopped. In the bottom row we scaled the mean intercept and slope curves to facilitate comparison of the curves.

Eye velocity typically increased rapidly with caloric stimulation, and would then plateau or decline modestly, until the irrigation stopped, and then velocity would drop rapidly (see bithermal example in Figure 1). For each data set, we fit straight lines to 30 seconds of data, and then shifted the time period every 5 seconds. The intercepts (Figure 2, top row) thus show how velocity at straight ahead evolves over time, and the slopes (Figure 2, middle row) show how the dependence of velocity on position changes with time. Nystagmus increased after stimulation began, peaking on average after 107 seconds (cold = 117 s, warm = 110 s, bithermal = 97 s). The change in velocity with eye position followed a similar time course, reaching minimums at 118, 119 s, and 113 s for cold, warm, and bithermal stimulation, respectively.

While nystagmus generally followed a smooth time course (Figure 2, top row), the fitted slopes were variable within and between subjects (Figure 2, middle row). Some of this variability was probably due to the difficulty subjects had in directing their gaze to the flashing target; in some trials subjects’ eye position did not change over as large a range (40°) as desired, so the fitted slopes show more variability as a result. In addition, some subjects seemed surprised and distracted when the sensation of motion first appeared, and they did not track the flashing target. If eye position remained in one direction as the nystagmus increased, the resulting slope estimate was biased. This led to increased variability in the slope estimates shortly after the onset of stimulation.

**Figure 3:**
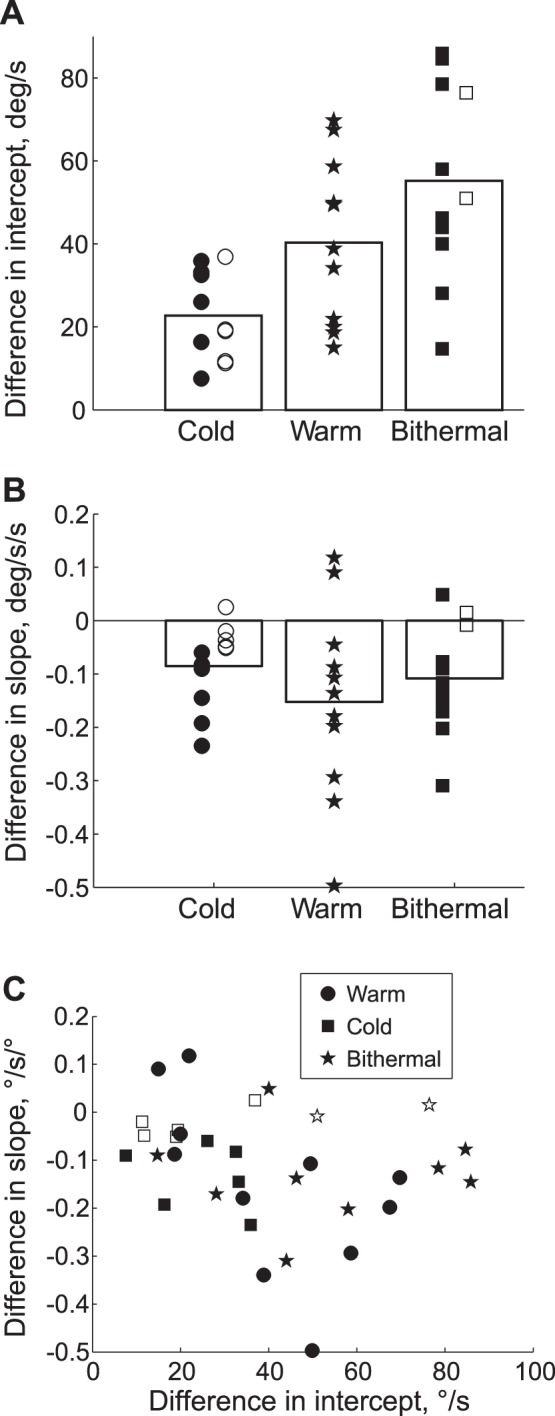
Linear regression of velocity on position at the time of peak nystagmus. A. Each point shows the difference in intercept from the control trial for an individual subject and the bars are means. Open symbols indicate the fitted *slopes* were not significantly different from control values. B. Each point shows the change in slope from the control trial for each subject and the bars are means. Open symbols indicate the difference in slopes from control values were not significantly different zero. C. The slopes (from panel B) are plotted against the intercepts (from panel A). Open symbols indicate the difference in *slopes* from control values were not significantly different from zero.

**Figure 4:**
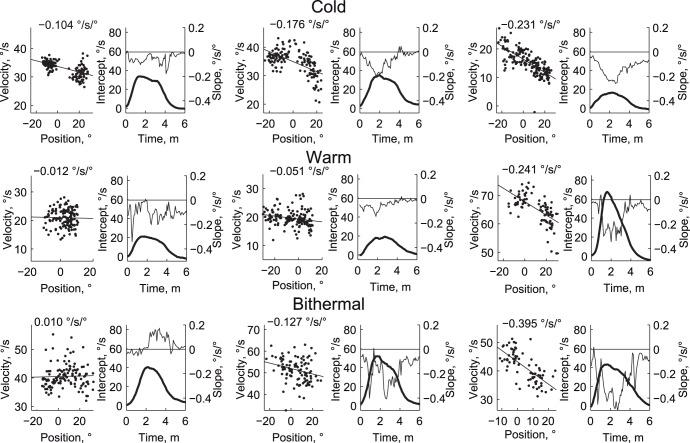
Example velocity versus position plots. Each row contains 2 pairs of plots, each pair showing the data from a different subject. The first plot in each pair is a scatter plot where each point shows the slow phase position and velocity from the 30 seconds centered on the time of maximum nystagmus velocity. The second plot in each pair shows the fitted intercepts (thick line, left-side axis) and slopes (thin line, right-side axis) for the first 6 minutes after the start of caloric stimulation. Each row shows a different stimulation condition (top = cold, middle = warm, bottom = simultaneous bilateral bithermal).

At the time of maximum velocity (the peaks in Figure 2, top row), the average difference in intercepts from control across subjects were 23°/s, 40°/s, and 55°/s for cold, warm, and bithermal stimulation, respectively (Figure 3A; see Figure 4 for example fits). The one way ANOVA was significant (F(2,20) = 24.3; p<0.001), and multiple comparisons found that cold stimulation produced significantly less intense nystagmus than both warm (p<0.001) and bithermal (p<0.001) stimulation, and bithermal stimulation produced marginally greater nystagmus than warm (p = 0.065).

At the time of maximum nystagmus, the average difference in slopes across subjects, from control, were −0.085, −0.152, −0.109°/s/° for cold, warm, and bithermal stimulation, respectively (Figure 3B). ANOVA did not find significant differences between the slopes (F(2,20) = 0.83, p = 0.45). With cold stimulation, 6 of 11 subjects had slopes that became more negative with stimulation, and 5 had no significant change. With warm stimulation, the slopes of 9 of 11 subjects became more negative, and 2 became significantly positive (a reversal of Alexander’s law). For bithermal stimulation 8/11 had slopes that became more negative, 1 became significantly positive, and 2 had no significant change. In each condition, the average difference of slope was significantly different from zero (all ps<0.02) An integrator time constant can be inferred from the fitted equations by −1/slope; average time constants were thus about 12, 5, and 8 seconds for cold, warm, and bithermal stimulation, respectively. By way of comparison, an average time constant of 52 seconds was found for control data analyzed with simple linear fits.

The average slopes and intercepts followed similar time courses (Figure 2, bottom row). The time courses were compared by fitting lines to adjacent 16 second blocks of data, and cross correlating the fitted slopes and intercepts when the velocity was more than 10% of the maximum velocity. In 16 of 33 experiments the correlations were significant (5 cold, 7 warm, 4 bithermal) with average correlations of −0.43. On average, the intercept lagged the slope by 17 seconds, which was not significantly different from zero (t = 1.0, p<0.3). ANOVA did not find significant differences for the lag times for the different conditions (F(2,20) = 2.3, p>0.1). The lag of the intercept is most obvious in the cold condition when the nystagmus was declining, showing that the eye position effect tended to recover to normal slightly faster than the nystagmus decayed. Repeating the cross correlation for the data after the peak nystagmus velocity found an average correlation of −0.44, which was significant in 16 of 33 experiments (4 cold, 7 warm, 5 bithermal), and the average lag of the intercept was 48 seconds (t = 2.9, p = 0.006). In the cold condition, the intercept lagged by 84 seconds (t = 2.5, p = 0.03), in the bithermal condition the mean lag of the intercepted was 48 seconds (t = 2.1, p = 0.059), and in the warm condition the mean lag was 11 seconds (t = 0.4, p = 0.6). Correlations for the data when nystagmus was rising were less strong overall (significant in only 7 of 33 experiments, with a mean of −0.45), and the mean lag was 3 seconds, which was not different from zero (t = 0.3, p = 0.7). (Note that because nystagmus rose faster than it decayed, there was less data entering into these correlations.).

Focusing on the time of maximum nystagmus, there was a modest correlation between the nystagmus velocity and the slope (Figure 3C). Overall, the correlation was not quite significant (Spearmann’s rho =  −0.33, p = 0.06), though the correlation for warm stimulation was significant (rho = −0.655, p = 0.034).

### Second Order Fits

We fit parabolas to the data from 2–3 minutes after the start of stimulation (when the target position shifted between 5 positions, instead of 2). While the quadratic component was often significant (5/10 cold, 4/10 warm, 4/10 bithermal), the sign of the components were not consistent, so the average quadratic components were not different from zero.

### Eye Position Dependent Changes in Velocity Decline with the Decay of Vestibular Nystagmus

The fitted slopes returned towards control values as the nystagmus decayed (see Figure 2). When nystagmus velocity was higher, particularly with warm and bithermal stimulation, the direction of nystagmus could reverse a few minutes after stimulation stopped (after 2.8 minutes, on average). We did not always observe a reversal, though this could be because we stopped the experiment early. In 16 cases (3 cold, 6 warm, 7 bithermal) we could further analyze the data and so to characterize the reversal we fit straight lines to 30 seconds of data, centered on the peak reversal velocity. The average intercepts were 2.1°/s, 3.3°/s, and 3.7°/s for cold, warm, and bithermal stimulation, respectively. In 11/16 cases the fitted slopes were not significantly different from control. The slopes were more negative than control values in 4 cases (1 cold, 1 warm, 2 bithermal), that is, Alexander’s law was followed in the reversal period. In 1 bithermal case the slope was more positive, or a reversal of Alexander’s law.

## Discussion

We found that eye velocity depended upon position in accordance with Alexander’s law when it was induced by warm, cold, and simultaneous bilateral bithermal caloric stimulation. The average velocity to position slope (−0.11) is very similar to the slopes we found in patients (−0.088 and −0.1) [12], [13]. Our results provide little support for Robinson’s et al [2] hypothesis that Alexander’s law arises due to an adaptive response to un-physiologic stimulation. Bithermal stimulation mimics the ‘push-pull’ of normal vestibular stimulation, yet we usually observed Alexander’s law in this condition.

The time course of the change in velocity with position, on average, closely followed that of the speed of nystagmus. There was, however, considerable variability in the velocity-position slopes, which made it difficult to determine the onset of Alexander’s law, or any temporal delay of the rise of Alexander’s law with the nystagmus velocity. Robinson’s et al [2], in three subjects, reported that the time of peak Alexander’s law lagged the time of peak nystagmus by 25 seconds, on average. Given the variability we observed in the slopes, however, such a measurement does not seem informative here. We correlated the slopes and intercepts over several minutes, and found the optimal temporal lag from the cross-correlation was not significantly different from zero. Since we binned the data in 16 second blocks, this would suggest that any lag is less than half the bin width, or 8 seconds, which is less than the 25 seconds suggested by Robinson et al [2]. This leads us to question whether it is really an adaptive phenomenon, since oculomotor adaptive effects typically need many minutes to develop, though rapid adaptation is not unprecedented (eg., [15], [16]). Caloric stimulation, like peripheral vestibular disorders, has a frequency content that is much lower than that produced by natural head movements, and could explain why Alexander’s law has not been observed during higher frequency head rotations. [2], [8]. Perhaps very low frequency signals are not well integrated.

Our results seem to contradict those of Jeffcoat et al [17], who reported a ‘reversed’ Alexander’s law with warm calorics. They interpret this result as supportive of Doslak’s model of Alexander’s law [6], [7], which includes an eye-position dependency in the VOR pathway. However, in Supporting Information S1 we explain that this is a mis-interpretation of Doslak’s model. Nonetheless, the results of Jeffcoat et al [17] with warm calorics are different than ours. However, they used a protocol that likely produced very low levels of nystagmus (the head was upright, whereas the optimal position for caloric stimulation is 60° pitched backwards; their example figures show velocity around 10°/s, whereas our average peak velocity was 40°/s). In the two cases where we observed a reversal of Alexander’s law, the nystagmus velocity was quite low (see Figure 3C).

Our results also do not conclusively support the Doslak model. This model predicts that the eye-position dependent effect should be independent of the nystagmus speed (above a threshold), yet on average we find that the effect rises and falls with the nystagmus (see Figure 2 and Figure 3C). In addition, Doslak’s model predicts that Alexander’s law should be observed during normal head movements, yet this does not seem to be the case for head impulses [8] or 0.5 Hz frequency head rotations [2].

What other mechanisms might account for Alexander’s law? The translational VOR is highly dependent upon gaze direction, and backward head movements produce centripetal eye movements, which, if added to a rotational VOR signal would produce Alexander’s law. The sensitivity of extraocular motor neurons varies with gaze position when stimulated with high intensity auditory stimuli (clicks) [18], which stimulate the otoliths organs [19], [20], and Jeffcoat et al [17] proposed this as the basis of an explanation of Alexander’s law. However, Alexander’s law has not been found during high frequency head rotations [2], [8]. There is evidence that caloric stimulation evokes horizontal linear VOR responses due to central processing to resolve the conflict between the dynamic canal stimulus and the static otolith signal [21]. To produce Alexander’s law would require the target distance to vary (being nearer when in the fast phase direction since the gain of the linear VOR is higher for near targets). Similarly, the rotational VOR has been found to be modestly sensitive to vergence angle [22], [23]. In our setup subjects viewed (flashing) targets on a flat screen and so the eccentric targets were more distant than central targets, but there was no left/right asymmetry which would provoke different vergence responses, so a contribution of vergence to Alexander’s law seems unlikely. Thus, at this point the mechanism responsible for Alexander’s law remains unclear. Our data do not fully support either the neural integrator hypothesis of Robinson et al [2], or the VOR modification model of Doslak [6], [7], and a vergence mediated effect seems unlikely.

To explain our recent findings in patients with acute unilateral vestibular deficit, where slow-phase eye velocity varied nonlinearly with eye position [12], [13], we developed a new model for Alexander‘s Law [24]. The simultaneous disfacilitation of the ipsilesional and hyperactivity of the contralesional vestibular nuclei following a peripheral vestibular lesion introduces an asymmetry in the responses of bilateral vestibular nuclei [25]. We hypothesize that this central asymmetry limits the linear operating range of the central responses. We showed that this results in eye position dependent gains in the central positive feedback loops that perform integration of velocity commands. Therefore, the time constant of the neural integrator becomes dependent on eye position, and nonlinear velocity-versus-position plots result. We further speculate that a similar mechanism could be responsible for Alexander’s Law during calorics; a continuous low-frequency stimulation like calorics could in the same manner saturate or inhibit the central responses and influence the integrator in a matter of seconds.

We did not find consistent non-linear velocity-versus-position effects with caloric stimulation, leaving open the possibility that the effects found in patients might be the result of adaptive mechanisms to suppress nystagmus, since these patients typically had nystagmus for several days before they were measured. Thus, the patterns of eye position dependencies seen in patients could be the combination of two different mechanisms, an immediate linear effect as seen with caloric stimulation, and slower adaptive changes which produce the non-linear patterns. If changes in the neural integrator are responsible for these effects, it suggests the integrator time constant depends on gaze direction. Adaptation of the neural integrator time constant depending upon gaze direction has been suggest previously [26], [27], [28], and has also been proposed based on pharmacological inactivation studies in monkeys [29] and more recently in goldfish [30].

## Supporting Information

Figure S1 **Predictions of Doslak’s (1979) model for unilateral warm and cold calorics.** Top, for a unilateral right side excitation, Doslak’s model results in a vestibular induced velocity that is independent of eye position (the dashed line) plus an eye position dependent term (the dotted line). The sum is such that the absolute velocity is larger in the fast-phase direction (right) and smaller in the slow phase direction (left), as expected from Alexander’s law. Bottom, in the same manner, a unilateral right side inhibition produces eye position dependent velocity in accordance with Alexander’s law.
(EPS)

Supporting Information S1 (DOCX)

## References

[1] Alexander G (1912) Die Ohrenkrankheiten im Kindesalter. In: Schlossmann A, editor. Handbuch der Kinderheilkunde. Leipzig: Vogel. 84–96.

[2] Robinson DA, Zee DS, Hain TC, Holmes A, Rosenberg LF (1984) Alexander’s law: its behavior and origin in the human vestibulo-ocular reflex. Annals of Neurology 16: 714–722. 6441510 10.1002/ana.410160614

[3] Hess K (1982) Do peripheral-vestibular lesions in man affect the position integrator of the eyes? Neuroscience Letters Suppl. 10: 242–243.

[4] Robinson DA (1968) Eye movement control in primates. The oculomotor system contains specialized subsystems for acquiring and tracking visual targets. Science 161: 1219–1224. 5302604 10.1126/science.161.3847.1219

[5] Robinson DA (1975) Oculomotor control signals. In: Lennerstrand G, Rita P, editors. Basic mechanisms of ocular motility and their clinical implications. Oxford - New York - Toronto - Sydney - Braunschweig: Pergamon Press. 337–378.

[6] Doslak MJ, Dell’ Osso LF, Daroff RB (1979) A model of Alexander’s law of vestibular nystagmus. Biological Cybernetics 34: 181–186. 314822 10.1007/BF00336969

[7] Doslak MJ, Dell’Osso LF, Daroff RB (1982) Alexander’s law: a model and resulting study. Ann Otol Rhinol Laryngol 91: 316–322. 7092055 10.1177/000348948209100318

[8] Anagnostou E, Heimberger J, Sklavos S, Anastasopoulos D (2011) Alexander’s law during high-acceleration head rotations in humans. Neuroreport 22: 239–243. 21346643 10.1097/WNR.0b013e3283451769

[9] Oosterveld WJ, de Jong HA (1987) The caloric vestibular test in weightlessness. Arch Otorhinolaryngol 244: 155–158. 3675298 10.1007/BF00464260

[10] Paige GD (1985) Caloric responses after horizontal canal inactivation. Acta Otolaryngol 100: 321–327. 4082971 10.3109/00016488509126555

[11] Scherer H, Brandt U, Clarke AH, Merbold U, Parker R (1986) European vestibular experiments on the Spacelab-1 mission: 3. Caloric nystagmus in microgravity. Exp Brain Res 64: 255–263. 3492382 10.1007/BF00237741

[12] Bockisch CJ, Hegemann S (2008) Alexander’s law and the oculomotor neural integrator: three-dimensional eye velocity in patients with an acute vestibular asymmetry. Journal of Neurophysiolgy 100: 3105–3116. 10.1152/jn.90381.2008 18799600

[13] Hegemann S, Straumann D, Bockisch C (2007) Alexander’s law in patients with acute vestibular tone asymmetry–evidence for multiple horizontal neural integrators. J Assoc Res Otolaryngol 8: 551–561. 17879115 10.1007/s10162-007-0095-6 PMC2538344

[14] Becker W, Klein HM (1973) Accuracy of saccadic eye movements and maintenance of eccentric eye position in the dark. Vision Research 13: 1021–1034. 4713917 10.1016/0042-6989(73)90141-7

[15] Melvill Jones G, Guitton D, Berthoz A (1988) Changing patterns of eye-head coordination during 6 h of optically reversed vision. Exp Brain Res 69: 531–544. 3371436 10.1007/BF00247307

[16] Miller JM, Anstis T, Templeton WB (1981) Saccadic plasticity: parametric adaptive control by retinal feedback. J Exp Psychol Hum Percept Perform 7: 356–366. 6453929 10.1037//0096-1523.7.2.356

[17] Jeffcoat B, Shelukhin A, Fong A, Mustain W, Zhou W (2008) Alexander’s Law Revisited. Journal of Neurophysiolgy 100: 154–159. 10.1152/jn.00055.2008 18450584

[18] Zhou W, Xu Y, Simpson I, Cai Y (2007) Multiplicative computation in the vestibulo-ocular reflex (VOR). 2007/01/26: 2780–2789. 10.1152/jn.00812.2006 17251367

[19] Murofushi T, Curthoys IS (1997) Physiological and anatomical study of click-sensitive primary vestibular afferents in the guinea pig. Acta Otolaryngol 117: 66–72. 9039484 10.3109/00016489709117994

[20] Murofushi T, Curthoys IS, Topple AN, Colebatch JG, Halmagyi GM (1995) Responses of guinea pig primary vestibular neurons to clicks. Exp Brain Res 103: 174–178. 7615033 10.1007/BF00241975

[21] Peterka RJ, Gianna-Poulin CC, Zupan LH, Merfeld DM (2004) Origin of orientation-dependent asymmetries in vestibulo-ocular reflexes evoked by caloric stimulation. J Neurophysiol 92: 2333–2345. 15175373 10.1152/jn.00174.2004

[22] Paige GD, Telford L, Seidman SH, Barnes GR (1998) Human vestibuloocular reflex and its interactions with vision and fixation distance during linear and angular head movement. J Neurophysiol 80: 2391–2404. 9819251 10.1152/jn.1998.80.5.2391

[23] Crane BT, Demer JL (1998) Human horizontal vestibulo-ocular reflex initiation: effects of acceleration, target distance, and unilateral deafferentation. J Neurophysiol 80: 1151–1166. 9744929 10.1152/jn.1998.80.3.1151

[24] Khojasteh E, Bockisch CJ, Straumann D, Hegemann SCA (2012) A dynamic model for eye-position-dependence of spontaneous nystagmus in acute unilateral vestibular deficit. European Journal of Neuroscience:. 10.1111/ejn.12030 23106392

[25] Smith PF, Curthoys IS (1989) Mechanisms of recovery following unilateral labyrinthectomy: a review. 14: 155–180. 10.1016/0165-0173(89)90013-1 2665890

[26] Chan WW, Galiana HL (2005) Integrator function in the oculomotor system is dependent on sensory context. Journal of Neurophysiolgy 93: 3709–3717. 10.1152/jn.00814.2004 15703232

[27] Chan WW, Galiana HL (2007) A non-linear model of the neural integrator in oculomotor control. Conf Proc IEEE Eng Med Biol Soc 1: 1156–1159. 10.1109/IEMBS.2007.4352501 18002167

[28] Mensh BD, Aksay E, Lee DD, Seung HS, Tank DW (2004) Spontaneous eye movements in goldfish: oculomotor integrator performance, plasticity, and dependence on visual feedback. Vision Res 44: 711–726. 14751555 10.1016/j.visres.2003.10.015

[29] Crawford JD, Vilis T (1993) Modularity and parallel processing in the oculomotor integrator. Exp Brain Res 96: 443–456. 8299746 10.1007/BF00234112

[30] Aksay E, Olasagasti I, Mensh BD, Baker R, Goldman MS, et al (2007) Functional dissection of circuitry in a neural integrator. Nature Neuroscience 10: 494–504. 17369822 10.1038/nn1877 PMC2803116

