# Supplementary material for: Development of Eye Position Dependency of Slow Phase Velocity during Caloric Stimulation
Source: PLoS One. 2012 Dec 12;7(12):e51409. doi: 10.1371/journal.pone.0051409 (PMC3520909; doi:10.1371/journal.pone.0051409)
Supplement: Supporting Information S1 — (DOCX) [file pone.0051409.s002.docx]

Development of eye position dependency of slow phase velocity during caloric stimulation

Authors:

Christopher J. Bockisch, Elham Khojasteh, Dominik Straumann, Stefan C.A. Hegemann

# Supplementary material

Here we analyze the model of Doslak et al (1979) to show that it predicts Alexander’s law occurs with unilateral excitation (e.g. warm calorics in the right ear) in exactly the same way as unilateral inhibition (cold calorics in the right ear) does. All notations below are taken exactly from Doslak’s model [[6](#_ENREF_6)]. Also, according to their reference frame, rightward eye movements are considered positive in the following analyses.

Let us first assume that there is a right side excitation such that:$V_{R}-V_{L}=V_{0}$ and $V_{0}\geq V>0$, where *V* is the threshold (*gating voltage*) that should be exceeded to activate the switch. Hence the *desired gaze* signals will pass through to the left side but not to the right side. So, R and L signals in the model will be:

$R=V_{L}-V_{R}=-V_{0}$ (1)

$L=(V_{R}-V_{L})+k(G-T)=V_{0}+k(G-T)$ (2)

where: $G=G_{R}-G_{L}$, *G_R_* and *G_L_* are right and left desired gaze signals, T is a constant tone signal, and *k* is an attenuation constant. Given zero initial conditions, eye position θ*_E_* and velocity θ′*_E_* will be:

$\theta_{E}=\frac{R-L}{s}=\frac{-V_{0}-V_{0}-k(G-T)}{s}=-\frac{2V_{0}}{s}-\frac{k(G-T)}{s}$ (3)

$\theta'_{E}=(-2V_{0}+kT)-kG$ (4)

where *s* is the complex frequency. Equation 4 is demonstrated in Figure S1A, where the dashed line is the first term in (4) (pure vestibular velocity) and the dotted line is the second term (gaze dependent), and the thick line shows the sum of the two. So, for a fixation point on the right side (fast-phase direction) the *absolute* velocity is larger, in accordance with Alexander’s Law. In the same manner, for a right side deficit (right side inhibition or cold calorics), the gaze dependent signals will pass through to the right side but not to the left. We can show that:

$\theta'_{E}=(+2V_{0}-kT)-kG$ (5)

This equation is also demonstrated in Figure (S1B). Therefore, in both cases, the model produces Alexander’s law.

So in summary, in the Doslak (1979) model, an eye-position dependent command is added to the vestibular signal whenever the canal asymmetry exceeds a threshold. Whether the eye position dependent velocity is higher on the left or right side depends upon which canal produces a greater signal, but how this difference arises is irrelevant in the model. So, for example, a left sided inhibition (cold calorics) produces the same asymmetry pattern and eye-position dependent signal as right sided excitation (warm calorics), and the model predicts eye velocity will be higher in the fast-phase direction in each case.
